# Structural properties of apolipoprotein A-I mimetic peptides that promote ABCA1-dependent cholesterol efflux

**DOI:** 10.1038/s41598-018-20965-2

**Published:** 2018-02-13

**Authors:** Rafique M. Islam, Mohsen Pourmousa, Denis Sviridov, Scott M. Gordon, Edward B. Neufeld, Lita A. Freeman, B. Scott Perrin, Richard W. Pastor, Alan T. Remaley

**Affiliations:** 1 School of Systems Biology, George Mason University, Fairfax, VA 22030 USA; 2 Cardiovascular and Pulmonary Branch, National Heart, Lung and Blood Institute, National Institutes of Health, Bethesda, MD 20892 USA; 3 Laboratory of Computational Biology, National Heart, Lung and Blood Institute, National Institutes of Health, Bethesda, MD 20892 USA

## Abstract

Peptides mimicking the major protein of highdensity lipoprotein (HDL), apolipoprotein A-I (apoA-I), are promising therapeutics for cardiovascular diseases. Similar to apoA-I, their atheroprotective property is attributed to their ability to form discoidal HDL-like particles by extracting cellular cholesterol and phospholipids from lipid microdomains created by the ABCA1 transporter in a process called cholesterol efflux. The structural features of peptides that enable cholesterol efflux are not well understood. Herein, four synthetic amphipathic peptides denoted ELK, which only contain Glu, Leu, Lys, and sometimes Ala, and which have a wide range of net charges and hydrophobicities, were examined for cholesterol efflux. Experiments show that ELKs with a net neutral charge and a hydrophobic face that subtends an angle of at least 140° are optimal for cholesterol efflux. All-atom molecular dynamics simulations show that peptides that are effective in promoting cholesterol efflux stabilize HDL nanodiscs formed by these peptides by the orderly covering of the hydrophobic acyl chains on the edge of the disc. In contrast to apoA-I, which forms an anti-parallel double belt around the HDL, active peptides assemble in a mostly anti-parallel “picket fence” arrangement. These results shed light on the efflux ability of apoA-I mimetics and inform the future design of such therapeutics.

## Introduction

Cholesterol homeostasis is critical for cell function, and a wide variety of regulatory pathways are involved in the maintenance of cholesterol at optimum levels^1–4^. One such pathway, the efflux of excess cellular cholesterol to highdensity lipoproteins (HDL), is mediated by several transporters, including ABCA1. Genetic defects in ABCA1 lead to Tangier Disease, which is characterized by the accumulation of cholesteryl esters in peripheral cells, particularly macrophages^5^. Efflux of excess cellular cholesterol is beneficial in reducing lipid accumulation in atherosclerotic plaques and hence several therapeutic strategies have been explored for enhancing this pathway^6^. HDL infusion therapy is one of these strategies^7^. Recombinant and/or purified apoA-I, the main protein component of HDL, is combined with phospholipids to produce reconstituted HDL (rHDL) particles that interact with several membrane proteins, such as the ABCA1 transporter, to efflux cholesterol. Infusion of rHDL increases the overall rate of cholesterol efflux from peripheral cells in both animal models and in humans^8–15^. HDL infusion rapidly decreases plaque lipid content in multiple animal models, and was effective in reducing plaque volume in early stage clinical trials of patients with Acute Coronary Syndrome^16–18^.

ApoA-I is a tandem array of amphipathic helices, the structural motif that appears necessary for apoA-I to remove cholesterol from cells by the ABCA1 transporter^19,20^. The exact molecular details of the cholesterol efflux process are not known but are thought to involve a process in which ABCA1 translocates lipids to the exofacial side of the plasma membrane. This generates a pleat of a lipid microdomain on the cell surface, which is then wrapped by apoA-I to produce nascent discoidal HDL^19^. There have been several structural models describing the arrangement of apoA-I around nascent discoidal shaped HDL. In the “picket fence” model, which is no longer accepted, at least for native HDL, α-helical arrays form antiparallel helices perpendicular to the plane of discoidal HDL^21,22^. Polarized internal reflection infrared spectroscopy unambiguously support the “belt” model^23^, in which two apoA-I molecules form a planar ring around the nanodisc. All current models of apoA-I on nascent HDL are based on this “belt” arrangement.

As an alternative to recombinant or purified apoA-I, several groups have shown that small synthetic apoA-I mimetic peptides can be used to produce HDL-like particles that promote cholesterol efflux. The use of synthetic peptides as a therapy has potentially multiple advantages over the use of fulllength apoA-I, and at least two apoA-I mimetic peptides have been tested in clinical trials^24–28^. ApoA-I mimetic peptides containing amphipathic helices made with either all L or D amino acids were similar in their detergent-like properties and were equally potent in promoting cholesterol efflux by ABCA1^29,30^. Peptides made with a mixture of L and D amino acids, which interfere with the stabilization of helix formation by hydrogen bonding, were ineffective^30^. Furthermore, stabilizing helix formation of mimetic peptides with salt bridges^25^, hydrocarbon staples^31^, or the use of conformationally restrained amino acids like proline^18^ have all been shown to increase cholesterol efflux^18,25,31,32^. However, some mimetic peptides, and especially bi-helical ones with very high hydrophobic moments, are cytotoxic and non-selectively remove cholesterol from cells^32^.

Despite the large body of research on apoA-I mimetic peptides, the role of amphipathic helices in cholesterol efflux and in their detergent-like properties is not well understood. Moreover, although the cholesterol-efflux functionality of apoA-I and mimetic peptides is similar, it is not known how similar their configurations are on a HDL-like particle. These questions are addressed in this study by comparing the structural properties and cholesterol efflux potential of four apoA-I mimetic peptides. A series of helical peptides comprised only of glutamic acid (Glu = E), leucine (Leu = L), and lysine (Lys = K) and sometimes alanine (Ala = A), with varying degrees of amphipathicity and net charge (hence the generic name ELK) were structurally characterized and tested for ABCA1-dependent cholesterol efflux. Next, molecular dynamics (MD) simulations of ELKs bound to the phospholipid surface and the edge of a nanodisc were carried out to reveal in atomistic detail the interactions of the ELKs with themselves and with membranes and their orientation on the nanodisc.

## Results

### ELK sequences, physiochemical properties, and conformations

**Figure 1:**
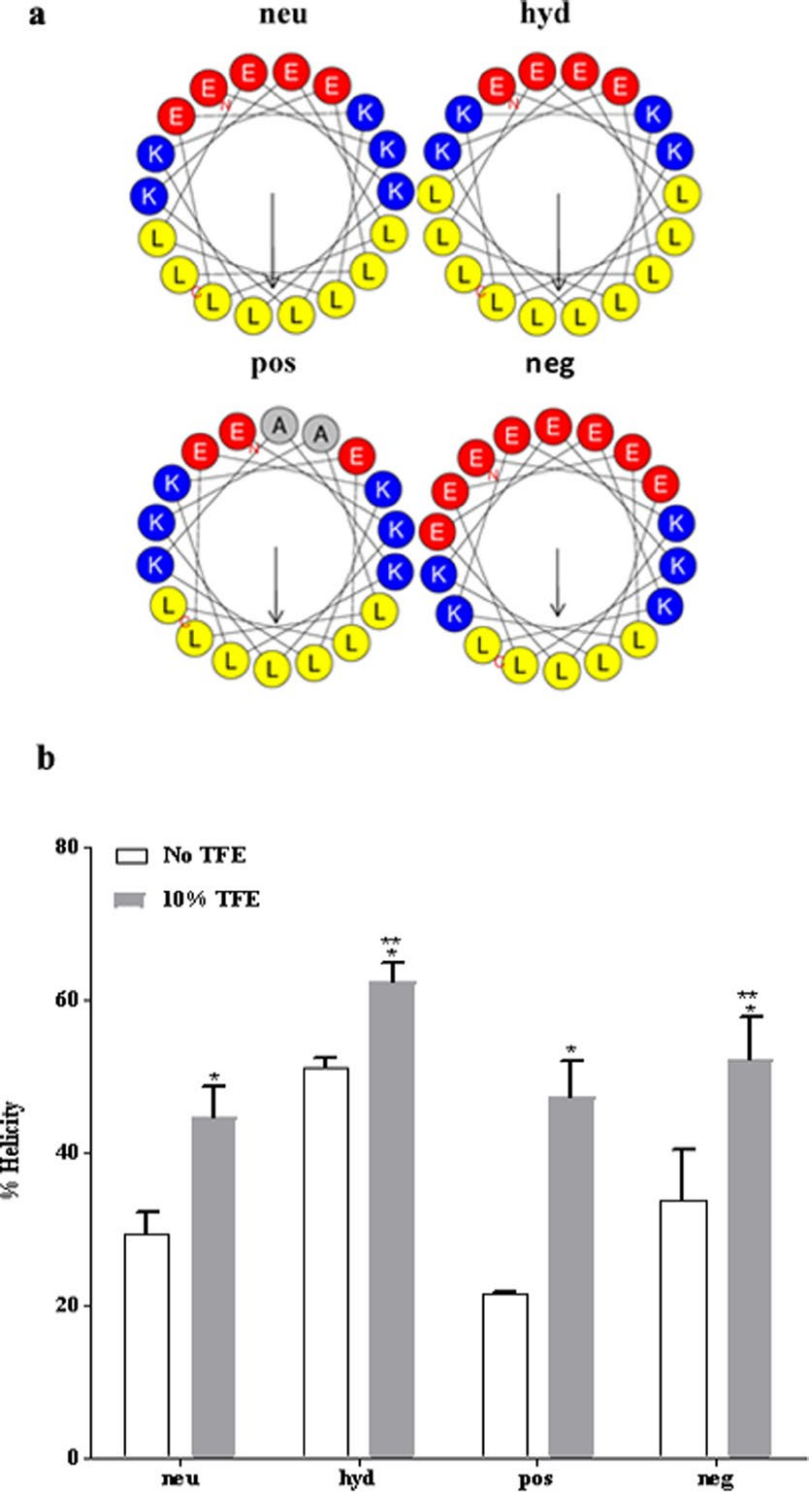
Physical properties of the ELKs. (**a**) Helical wheel diagrams of neu (EKLKELLEKLLEKLKELL), hyd (EKLLELLKKLLELLKELL), pos (EKLKALLEKLKAKLKELL), and neg (EELKEKLEELKEKLEEKL). Amino acids are shown as follows: Glu (E), red; Lys (K), blue; Leu (L), yellow; Ala (A), gray. Arrows are proportional to hydrophobic moments. (**b**) CD Spectroscopic analysis of the peptides. Percent helicity determined by circular dichroism (CD) spectroscopy. Measurements were carried out separately in an aqueous buffer (1% acetonitrile) plus minus TFE (10% trifluoroethanol). The mean residue ellipticity at 222 nm was used to calculate α-helicity. Statistical analysis was done using multiple t test. A global analysis for all peptides was performed in two buffer systems by using two-tailed unpaired t test. P values < 0.05 = *. Additionally, a comparison of all peptides against neu in 10% TFE was also performed using paired t test. p values are presented as p < 0.05 = **.

The primary amino acid sequences and physical properties of the four ELKs considered here are listed in Table 1. Each peptide is named based on its predominant characteristic: neutral (neu); hydrophobic (hyd); positive (pos); and negative (neg). Like other apoA-I mimetic peptides, neu was designed to form a classic Type A amphipathic helix^33^; i.e. comparably sized hydrophobic and hydrophilic faces, and positively charged residues on the hydrophobic-hydrophilic boundary (Fig. 1a). The hydrophobic face of all the ELKs contains Leu because of its favorable interaction with lipids^34^. The hydrophilic face contains both positively (Lys) and negatively (Glu) charged residues. The central part of the hydrophilic face of the ELKs contains negatively charged Glu, as is commonly found in amphipathic helices of apoA-I. Neu has an equal number of positively and negatively charged residues. The amino acids in hyd are arranged similarly to neu, but its hydrophobic face is larger. The third peptide, pos, has a net charge of +3 at pH 7.0 and a slightly smaller hydrophobic face than neu because of the replacement of one Leu with Lys and the replacement of two Glu with Ala. Finally, neg has a net negative charge of −3, because of an increased number of Glu residues, and the smallest hydrophobic face of the group.

**Table 1:** Physicochemical properties of ELKs. Wimley-White hydrophobicity indices^68^ were used for calculation of Mean Hydrophobicity and Hydrophobic Moment.

ELK	Sequence	MW	Iso-electric Point (pH)	Mean Hydrophobicity (kcal/mol)	Hydrophobic Moment (kcal/mol)	Net Charge (e)
neu	EKLKELLEKLLEKLKELL	2209	6.7	0.59	0.74	0
hyd	EKLLELLKKLLELLKELL	2178	6.7	0.36	0.70	0
pos	EKLKALLEKLKAKLKELL	2108	8.9	0.47	0.52	+3
neg	EELKEKLEELKEKLEEKL	2257	4.6	1.02	0.70	−3

**Figure 2:**
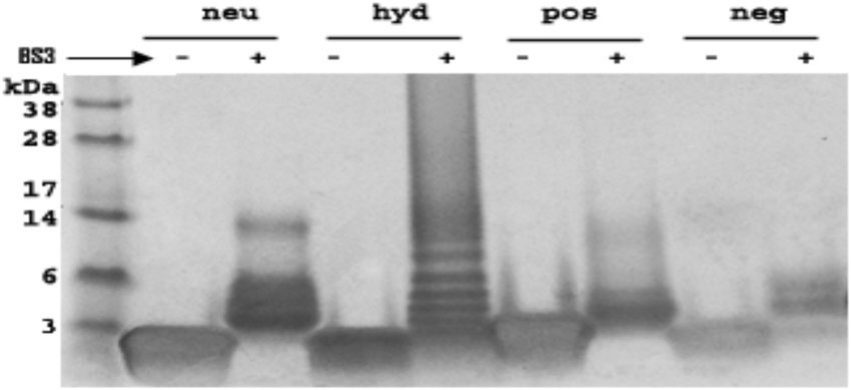
Cross-linking of ELKs. Cross-linking of ELKs with (+) or without (−) excess BS^3^ (bis[sulfosuccinimidyl] suberate) followed by SDS-PAGE (12% Tris-Tricine gel) electrophoresis. An uncropped image of the electrophoresis gel for Fig. 2 is shown in Supplementary Figure 1.

Figure 1b shows the results of circular dichroism (CD) spectroscopy, which was performed in either aqueous buffer [PBS + 1% acetonitrile (ACN); v/v] or the same buffer that also contained trifluoroethanol (TFE, 10%; v/v) to mimic a membrane environment^35^. All the peptides showed increased helicity in the presence of TFE. Hyd was the most helical peptide in both solvents. A potential explanation for these findings is that the peptides form oligomers in the aqueous buffer by the association of their hydrophobic faces similar to other apoA-I mimetic peptides^36,37^. This hypothesis was tested by cross-linking free peptides when dissolved in aqueous buffer. SDS-PAGE electrophoresis (Fig. 2 and Supplementary Figure 1) shows that both neu and hyd form oligomers containing up to 6 peptides, whereas pos and neg are mostly monomers and dimers, most likely because of charge repulsion.

### ELKs form lipoprotein-like particles in association with lipids

**Figure 3:**
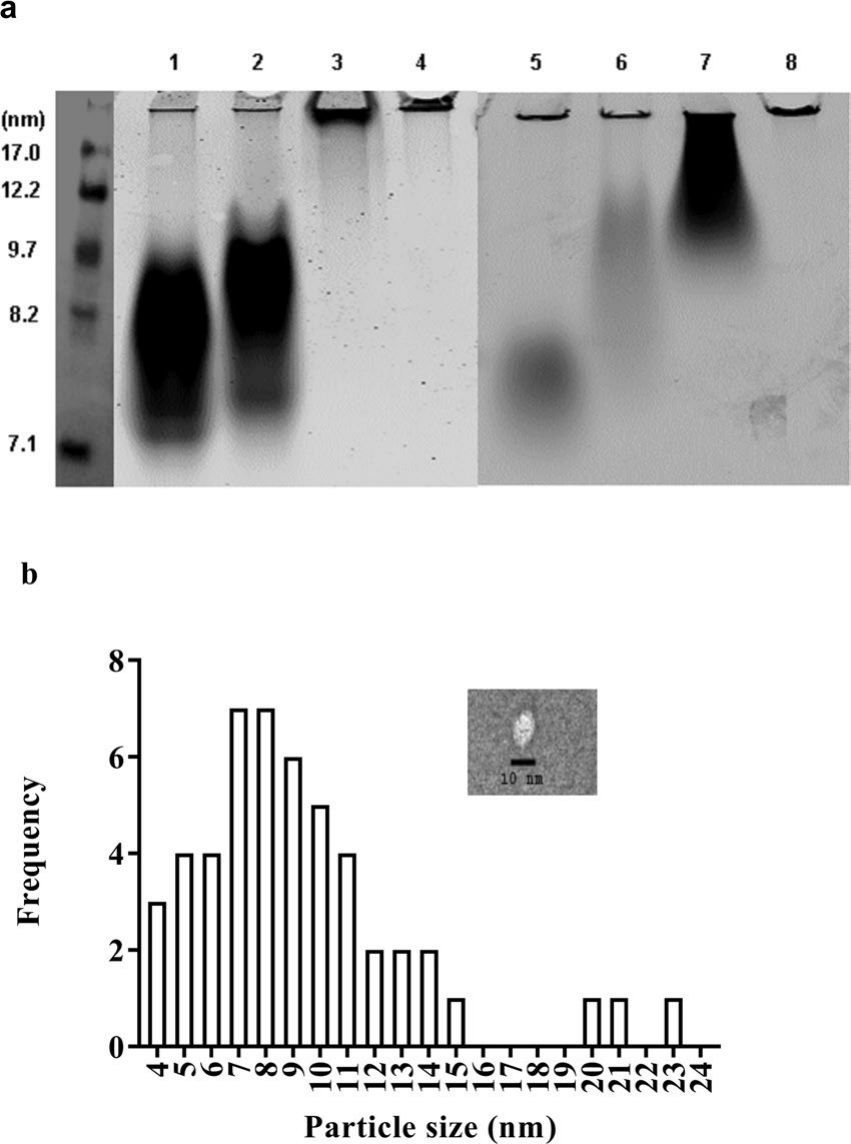
ELK-generated particle size by native gel electrophoresis and TEM. (**a**) Lipid particles generated by incubation of peptides with DMPC lipid vesicles (lanes 1 = neu, 2 = hyd, 3 = pos, 4 = neg) and a mixture of natural lipids found in cellular membranes (lanes 5 = neu, 6 = hyd, 7 = pos, 8 = neg). Both types of lipids were suspended in PBS at 1 mg/ml along with 0.5% PE-rhodamine. The vesicles were incubated with 0.5 mg/ml peptides in PBS at room temperature for 2 hours with gentle shaking. The lipid particles formed by peptides were separated in a 1D native TBE gel and scanned using a Typhoon scanner. (**b**) Particle size distribution after reconstitution of neu with phospholipid and cholesterol (neu:POPC = 1:6.25 and cholesterol:POPC = 1:10). Mean size of particles is 9.2 nm. Inset shows a TEM image of a representative particle made with neu. Scale 10 nm, magnification 50000X. Uncropped images of Fig. 3 are provided in Supplementary Figures 2, 3 and 4.

To be effective in promoting cholesterol efflux, apoA-I mimetic peptides must be able to extract cholesterol and phospholipids from cells and then stabilize them in aqueous solution by forming HDL-like particles similar to what apoA-I does after interaction with ABCA1^38^. As shown in Fig. 3a (lanes 1–4), both neu and hyd when combined with dimyristoylphosphatidylcholine (DMPC) and sonicated formed 7–9 nm sized particles similar in size to HDL. In contrast, no visible HDL-like size particles are observed with pos and neg. Also in Fig. 3a (lanes 5–8), the experiment was repeated, using a mixture of natural lipids, that are known to comprise lipid microdomains produced by ABCA1. Again, both neu and hyd form HDL size particles but the particles produced by hyd are considerably larger. Pos also forms particles about 12 nm in diameter, but again no particles are observed with neg. The particle size formed by neu was further investigated using Transmission Electron Microscopy (TEM), using 1-palmitoyl-2-oleoyl-phosphatidylcholine (POPC) with 6.25:0.625:1 POPC:chol:neu. The HDL-like particles formed were relatively homogenous with an average size of 9.2 nm (Fig. 3b and inset), which is comparable to that of a nascent HDL particle produced with apoA-I and also similar to particles produced by other apoA-I mimetic peptides^39^.

### ELKs solubilize lipids

**Figure 4:**
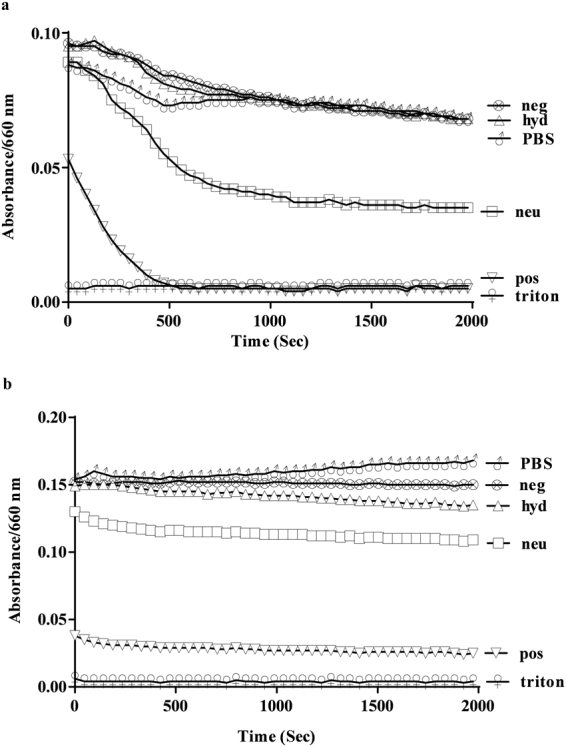
Lipid solubilization assay. ELKs dissolved in PBS or just PBS were continuously mixed with 0.1 mg/ml DMPC (**a**) and a mixture of natural lipids (**b**). Turbidity was monitored by measuring the absorption at 432 nm every 5 seconds for 50 min and changes were expressed relative to H_2_O. A sample with 1% Triton X-100 and an untreated sample were included in (**a**) and (**b**) as positive and negative control, respectively, and used to calculate % hemolysis.

The ability of the ELKs to act like peptide detergents by solubilizing phospholipids, a key feature for them to efflux cholesterol by ABCA1^1^, was tested by monitoring the turbidity of large multilamellar DMPC vesicles in the presence of the peptides. Similar to what has been shown for 5A, an apoA-I mimetic peptide^36^, pos readily dissolves DMPC vesicles and almost completely reduces the turbidity over 500 seconds (Fig. 4a). Neu also solubilized DMPC vesicles but is not as effective as pos. In contrast, hyd and neg are relatively inactive. Similar results are obtained with large multilamellar vesicles that are made with a lipid mixture that mimics the lipid microdomain of ABCA1 (Fig. 4b). Pos and neu almost immediately solubilize these vesicles, thus only a limited time-dependent decrease was observed. Hyd and neg were inactive with these vesicles as well.

### ELKs promote cholesterol efflux

**Figure 5:**
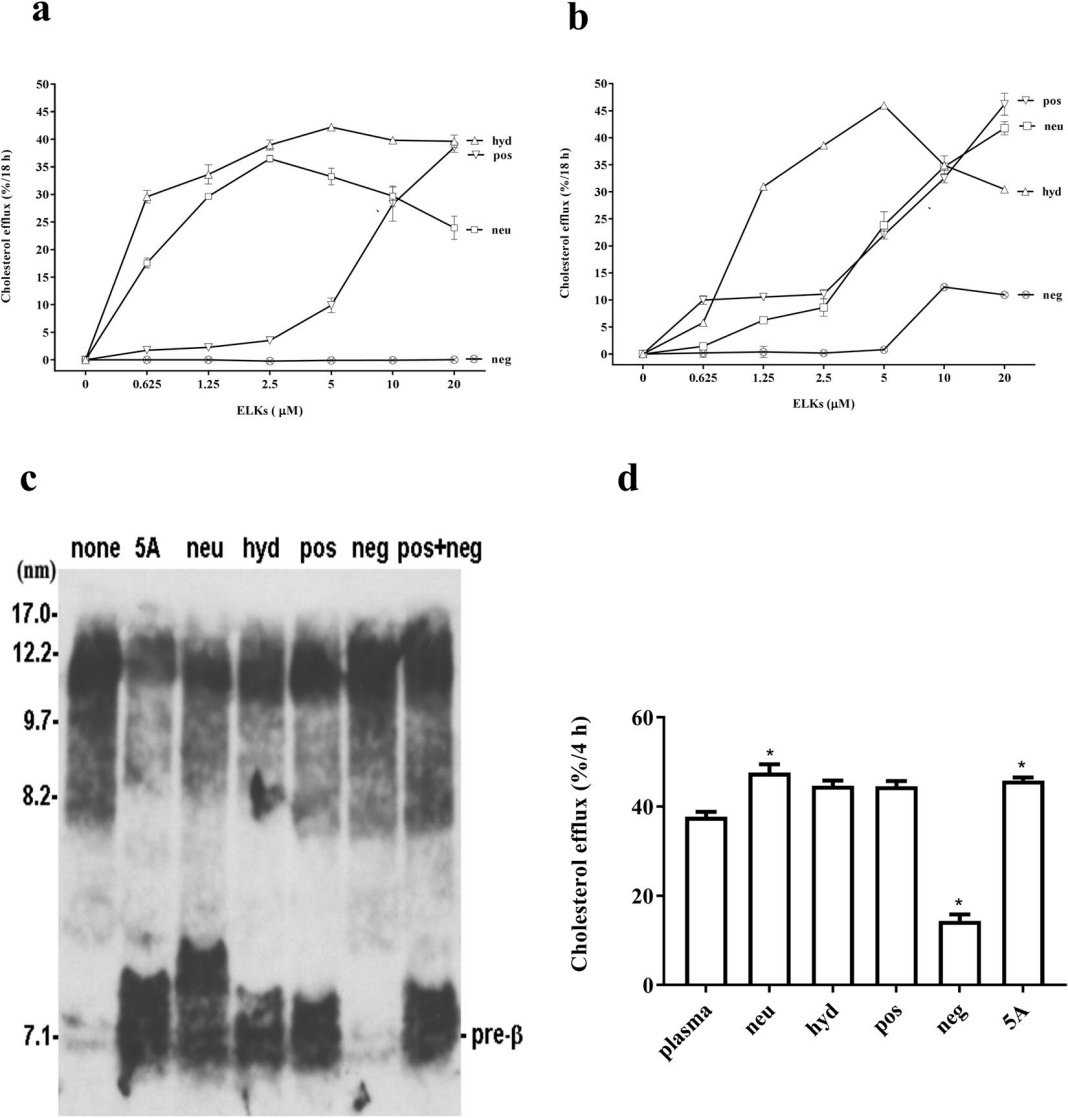
Efflux assay of ELKs. (**a**) Cholesterol efflux from ABCA1-BHK cells as a function of peptide concentration. (**b**) Cholesterol efflux from ABCA1 transporter induced in macrophages as a function of peptide concentration. Results are expressed as the mean ± 1 S.D. of triplicates in (**a**) and (**b**). A positive control using 0.5 µM apoA-I protein showed 30% (**a**) and 26% (**b**) efflux after 18 h. (**c**) Human plasma incubation of ELKs causes HDL remodeling. (**d**) Cholesterol efflux by ABCA1 transporter-expressing cells treated with human pooled plasma pre-treated with ELKs. A previously described apoA-I mimetic peptide, 5A, was included as a positive control (**c**) and (**d**). Statistical analysis was performed comparing each peptide with control sample (plasma alone) using paired t test and presented as p < 0.05 = *.

Next, the ability of ELKs to stimulate cholesterol efflux from a BHK cell line stably transfected with ABCA1 was tested. As Fig. 5a shows, both hyd and neu are effective in promoting cholesterol efflux and their apparent Vmax of 35–40%/18 h is similar to previously described apoA-I mimetic peptides^30,37,40^. Neu, however, is slightly less potent than hyd and at higher concentrations the rate of efflux appears to decrease, which has been previously described for other apoA-I mimetic peptides possibly due to either cytotoxicity or peptide aggregation^36^. Pos is also able to promote cholesterol efflux but at a much higher apparent Km (>8 μM). No evidence of cholesterol efflux was observed for neg at even the highest doses tested. None of the ELKs show significant cholesterol efflux from mock-transfected BHK cells (Supplementary Figure 5), thus showing their specificity for ABCA1. We expanded this study by also testing cholesterol efflux from J774 macrophages induced to express ABCA1 (Fig. 5b). Overall, the results were similar; hyd was the most effective, whereas neg was relatively inactive. The main difference from the transfected BHK cells is that neu was relatively less active in cholesterol efflux compared to hyd and yielded results similar to pos.

None of the peptides at the highest dose tested for the cholesterol efflux assay (20 µM) showed significant cell lysis when human red blood cells were treated with peptides (Supplementary Figure 7), which has been used as a sensitive measure of cytotoxicity for amphipathic peptides^36^. At 200 µM, pos and hyd showed some limited hemolysis but only lysed less than 4% of red blood cells.

In addition, we tested the effect of the addition of ELKs to plasma on pre-beta HDL formation and on cholesterol efflux (Fig. 5c). Like what has been described after the addition of apoA-I^41^, the addition of ELKs and the 5A peptide^39^ promoted the remodeling of HDL in plasma and caused the formation of pre-β HDL, which is known to be discoidal in shape and a potent stimulator of ABCA1-dependent cholesterol efflux^39^. Neg, however, did not increase the formation of pre-beta HDL and also did not appear to inhibit pre-β HDL formation when mixed with pos. All of the peptides except neg showed a small increase in cholesterol efflux from BHK-ABCA1 cells when added to plasma (Fig. 5d). Interestingly, neg inhibited cholesterol efflux when compared to plasma containing no peptides, but did not inhibit cholesterol efflux from BHK-ABCA1 cells when neg was mixed with pos (Supplementary Figure 8).

### MD simulations of interaction of ELKs with lipids

To gain insight into why ELKs differ in their interaction with lipids and in their ability to stimulate ABCA1-dependent cholesterol efflux, four sets of all-atom MD simulations were carried out.

**Figure 6:**
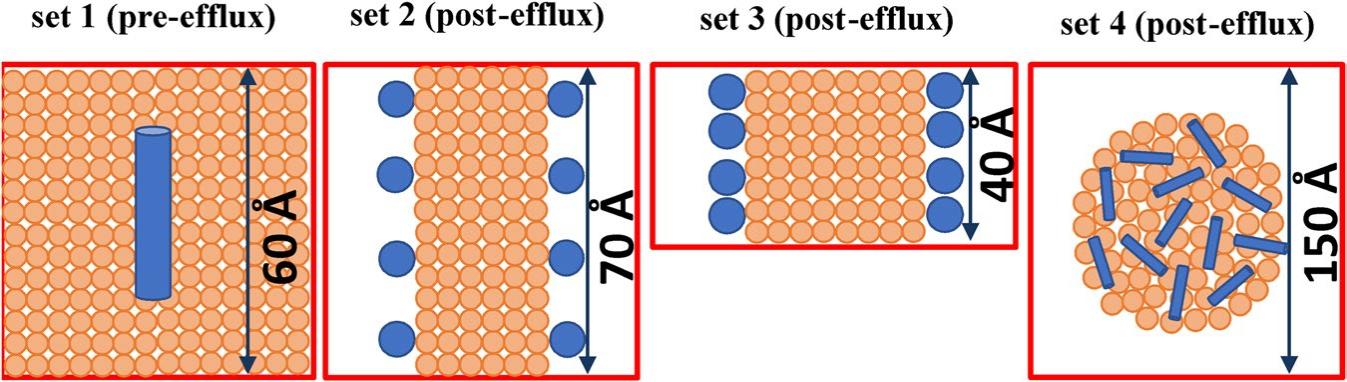
MD simulations of ELKs. Schematic top-down view of initial conditions in Set 1 (surface-bound), Set 2 (edge-bound, low density), Set 3 (edge-bound, high density), and Set 4 (nanodisc). Lipids, peptides, and box boundaries are shown in orange, blue, and red, respectively. Peptides are represented as cylinders (when perpendicular to bilayer normal) or circles (when parallel to bilayer normal). Boxes of Set 1, 2, and 4 are cubic. Box of Set 3 is tetragonal. All systems are hydrated with water and NaCl.

Set 1 aimed at understanding the pre-efflux events when peptides first bind to the surface of lipid domains on the plasma membrane generated by ABCA1. A hydrated bilayer model was created consisting of 90 POPCs and 10 cholesterols that interacted with two identical α-helical peptides, one per leaflet, placed above the C2 atoms of oleoyl acyl chains with their hydrophobic residues facing the bilayer interior (Fig. 6, leftmost panel). Peptides lie flat on the bilayer surface with varying degrees of insertion. As supplementary Figure 9 and Table 2 show, the insertion depth is ranked as hyd > neu > pos > neg, which is in the same order as the ability of peptides to promote ABCA1-dependent cholesterol efflux (Fig. 5). The large hydrophobic faces of hyd, neu, and pos enable them to reside between phosphate groups and C2 atoms of oleoyl acyl chains. In contrast, neg, which is the least active in cholesterol efflux, resides above the phosphate groups and is the most superficially bound of all peptides.

**Table 2:** Simulation details and results.

Simulation Type	POPC:chol:Peptide	Peptide	Analysis Time (ns)	Peptide Height (Å)	Number of Salt Bridges per peptide	
					Lys-Phos	Glu-Choline
Set 1 surface-bound	90:10:2	Neu	400	2.2 ± 0.1	3.9 (73)	1.3 (13)
		Hyd	400	0.7 ± 0.1	2.7 (57)	1.6 (15)
		Pos	400	3.3 ± 0.1	5.0 (73)	0.9 (13)
		Neg	400	6.4 ± 0.3	3.4 (78)	2.7 (15)
Set 2 edge-bound low density	80:8:8	Neu	400	NA	1.0 (40)	0.3 (6)
		Hyd	400	NA	0.7 (24)	0.3 (6)
		Pos	400	NA	1.9 (35)	0.3 (6)
		Neg	400	NA	1.3 (37)	0.6 (6)
Set 3 edge-bound high density	60:6:8	Neu	470	NA	0.5 (92)	0.2 (9)
		Hyd	470	NA	0.1 (41)	0.2 (7)
		Pos	470	NA	1.2 (50)	0.3 (11)
		Neg	470	NA	0.9 (130)	0.5 (10)
Set 4 nanodisc	150:15:24	Neu	500 ns	NA	0.7	0.2

Peptide height is defined as the vertical distance between peptides and C2 atoms of oleoyl acyl chains, and is not applicable (NA) to edge-bound simulations. Salt bridges were measured as the number of contacts between nitrogen of amino group of Lys and phosphorus of phosphate group of POPC, and carbon of carboxyl group of Glu and nitrogen of choline group of POPC, averaged over all simulation frames and divided by the number of peptides. Average lifetimes (in ps) are presented in parentheses. Lifetimes for Set 4 are not presented because the trajectory was saved every 240 ps, which is more than typical lifetimes

Because the positions of peptides are different, so are their interaction patterns with surrounding lipids, as evident from salt bridges in Table 2. For example, neg, the most superficially bound peptide, had the most Glu-choline salt bridges. In general, lifetimes of Lys-phosphate salt bridges are consistently higher than those of Glu-choline salt bridges. This is expected from Type A amphipathic peptides, whose negatively charged sidechains face water rather than adjacent lipids. The ability of peptides to form total salt bridges (Lys-phosphate plus Glu-choline) is ranked as neg > pos > neu > hyd and is in the reverse order of their ability to promote ABCA1-dependent cholesterol efflux.

Sets 2 and 3 were designed to investigate post-efflux events and test the hypothesis that peptides that best stabilize the discoidal bilayer form of nascent HDL would be the most efficient in cholesterol efflux. A patch of a nanodisc was modeled by introducing a water slab perpendicular to the bilayer head group surface, leading to acyl chains of edges exposed to water. Hence, the bilayer was exposed to water from two edges and two head group surfaces and was periodic along the third direction. See Fig. 6 (middle two panels) for a sketch of geometry.

In Set 2, the bilayer consisted of 80 POPCs, 10 cholesterols and a relatively low peptide density (8 per bilayer) to allow freedom of motion of the peptides. Four peptides were oriented parallel (head-to-head) to each other on one edge and four peptides antiparallel (head-to-tail) on the other edge of the bilayer, with their hydrophobic residues facing the bilayer acyl chains. The edges were approximately 70 Å long, thus large enough to allow for rearrangement of peptides. Side views of the two edges at 500 ns are presented in Supplementary Figure 10. Because the number of peptides per edge length is small, in all the systems the lipids tilt to shield their acyl chains from water, and concomitantly, peptides recruit them to form salt bridges.

**Figure 7:**
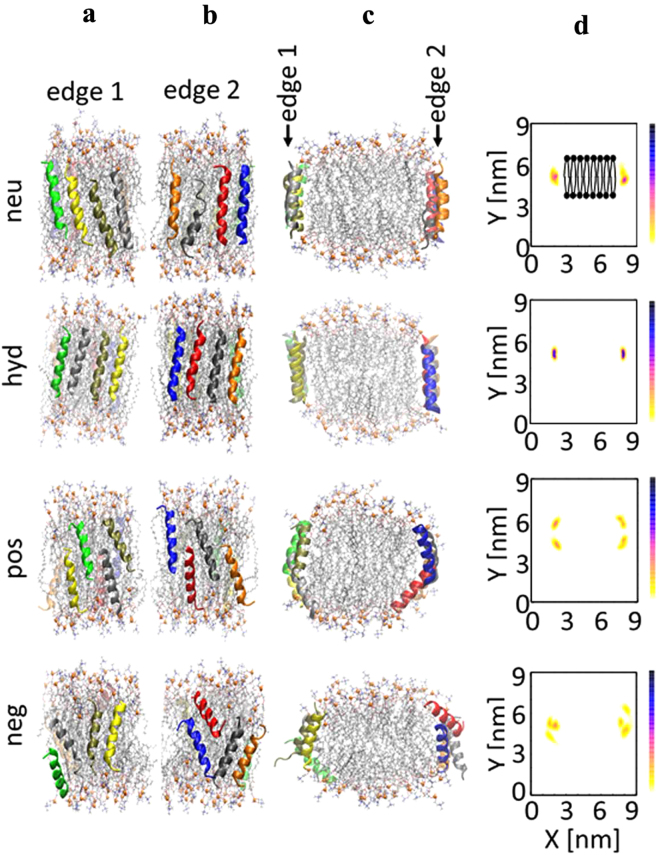
Four edge-bound simulations with 60:6:8 POPC:chol:peptide (Set 3, high density). Each system includes eight identical peptides located on two edges of a bilayer slab antiparallel to each other. (**a**–**c**) Side views at 500 ns. Side view along the bilayer periodicity is shown in (**c**). Peptides, colored ribbons; POPC, sticks; phosphorus of POPC, orange balls; cholesterols, black sticks. Schematic top-down view is shown in Fig. 6. (**d**) Number-density maps of C_α_ of 9^th^ residue of peptides viewed along the direction of bilayer periodicity. Bilayer is shown schematically in the top panel.

The simulations revealed the dimerization of the peptides when bound to bilayer edges. The dimers found here have at least one salt bridge or hydrophobic contact between two peptides. Because each edge contained either parallel or antiparallel arrangement of peptides, the differences in parallel and antiparallel dimers could be assessed. Due to repulsion of charged sidechains, the ELKs in parallel dimers were always partially mismatched; in contrast, antiparallel dimers formed with partially mismatched or fully matched peptides. As listed in Supplementary Table 1, hyd and neg each formed one parallel and two antiparallel dimers. Neu formed two parallel and two antiparallel dimers, although antiparallel dimers were more stable, because the number of intermolecular hydrophobic contacts were higher than that of parallel dimers. Pos only formed an antiparallel dimer, whereby Lys15 of a peptide salt bridged with the negatively charged C-terminus of the other. The simulations thus indicate that antiparallel dimers are more favored than parallel ones. Additionally, hyd can dimerize even without salt bridges and solely through hydrophobic interactions. As shown in Fig. 7, an antiparallel trimer of hyd is formed in which the peptides rendered in blue and red interact through hydrophobic effects and those in red and black interact through four salt bridges. The large hydrophobic face of hyd enables the blue peptide to twist and face both hydrophobic face of the red peptide and POPC acyl chains.

Set 3 differs from Set 2 in that only antiparallel dimers, taken from the last configurations of Set 2, were placed close to acyl chains of a smaller bilayer slab, hence high density edge-bound simulations (Fig. 6). The aim of this set was to contrast the abilities of peptides to cover the edge of a nanodisc patch at a density similar to the reconstituted HDL particles made with the peptides. The 500 ns snapshots of each simulation (Fig. 7) show consistent arrangements of peptides on the two edges. As demonstrated by number-density maps of peptides and lipid head groups in Fig. 7d and Supplementary Fig. 11, there is a distinct difference between the behavior of peptides and lipids in systems containing pos and neg and systems containing neu and hyd. Pos and neg migrate toward bilayer head groups and reside on the curved edge-surface boundaries, and concurrently, edge lipids tilt so their head groups face water and their hydrophobic acyl chains are more shielded in this configuration. In contrast, neu and hyd remain on the edges, and edge lipids remain oriented along the bilayer, and thus the hydrophobic acyl chains of the phospholipids are more covered by these peptides than by either pos or neg. Neu is slightly more scattered than hyd on edges as revealed by density maps, and thus, hyd is the most stable of peptides on edges, which is consistent with the superior cholesterol efflux property of this peptide.

**Figure 8:**
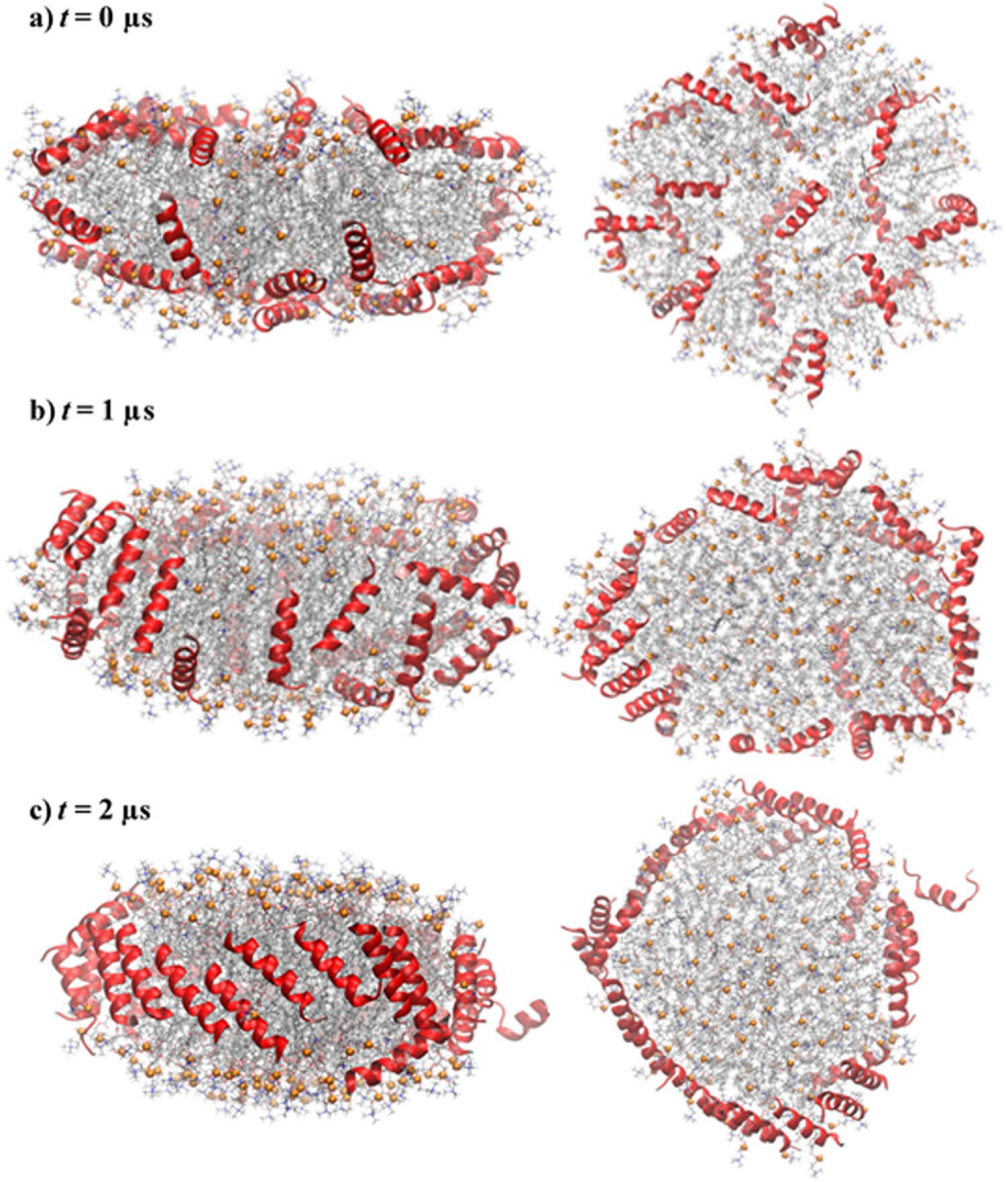
Picket-fence arrangement of neu on a HDL nanodisc. Stoichiometry of phospholipids, cholesterol and peptides was the following: 150:15:24 POPC:chol:neu. Left panels, side views; right panels, top-down views. (**a**) *t* = 0 μs. Peptides are initially placed on disc surface randomly. (**b**) *t* = 1 μs. 14 out of 24 peptides migrated to disc edge. (**c**) *t* = 2 μs. Peptides are initially arranged in a “picket-fence” orientation. Peptides are shown in *red* ribbons. POPC and cholesterol are represented as sticks: acyl chain, *silver*; nitrogen, *blue*, oxygen, *red*; phosphorus, *orange* balls; cholesterol carbons, *black*.

In Set 4, an entire nanodisc with 150:15:24 POPC:chol:neu was simulated for 2 µs on the Anton-2 supercomputer to investigate the stability and orientation of neu on the edge, and test the reliability of the starting peptide placement in the slab model in Sets 2 and 3 (Fig. 6). This time the initial configuration was developed by randomly covering the surface of a nanodisc with peptides (see Methods and Fig. 8a). While this arrangement is not a stable one for the nanodisc (the hydrophobic edge is exposed to water), it allowed an unbiased evolution of the peptide assembly, but required relatively long computation time. One of the neu peptides escaped after 20 ns and never fully associated with the disc. By 1 µs, 14 out 24 neu peptides migrated to disc edge (Fig. 8b). At 2 μs, 20 neu peptides were at the edge, 3 at the edge-surface boundary, and 1 in water (Fig. 8c). They formed five antiparallel and three parallel dimers, thus showing the co-existence of the two dimer types and supporting the conclusion of Set 2 where antiparallel neu dimers were slightly more favored. In contrast to the antiparallel double belt arrangement well-established for apoA-I^21,22^, all but two peptides (one of which is in water) arranged as a “picket-fence” on the disc edge.

The disc diameter in Set 4 was obtained by orienting the disc normal along the *z* axis and comparing the *z*-component of moment of inertia of the disc with that of a homogeneous disc, *I*_*z*,disc_ = 1/2 *N R*^2^, where *R* and *N* are radius and number of atoms, respectively. Time series is presented in Supplementary Figure 12 and the average diameter between 1.5–2 µs is 93.5 ± 0.2 Å, consistent with the experimentally determined diameter of 92 Å. Each neu formed 0.7 salt bridges with phosphate groups, comparable to those obtained in Set 2 (1.0) and Set 3 (0.5) (Table 2).

## Discussion

Using a combination of biochemical and MD simulation studies, the structural and physical features of four different apoA-I mimetic peptides that are important in the formation of HDL-like particles and in the promotion of cholesterol efflux by the ABCA1 transporter were investigated. As the primary structural motif of apoA-I is amphipathic α-helix^42^, the four types of amino acids used to make the ELKs (E, L, K and A) were arranged to form such helices, which was confirmed by CD spectroscopy (Fig. 1b).

Pos is the most effective in solubilizing both synthetic DMPC and the mixture of natural phospholipid vesicles (Fig. 4), but unlike Neu and Hyd could only form relatively large HDL-like particles when co-sonicated with a mixture of natural lipids (Fig. 3). Neu had an intermediate ability in the vesicle solubilization assay, whereas hyd and neg were inactive. The likely reasons for superior vesicle solubilization ability of pos are 1) it has a large enough hydrophobic face to reside below phosphate groups of lipids where it can simultaneously maintain hydrophobic interactions with the membrane interior and form salt bridges with phosphate groups (Fig. 2 and Supplementary Figure 9), and 2) it has the highest number of Lys residues to top the other peptides in forming salt bridges with phosphate groups (Table 2). Neg and neu form comparable salt bridges when bound to the membrane surface. However, their insertion depths and experimentally-observed solubilization abilities are very different. Therefore, neg fails at solubilizing lipids because its small hydrophobic face prevents its deep insertion into the membrane, thereby resulting in a poor peptide-membrane hydrophobic interaction and weak binding. This also likely accounts for the inability of this peptide to form HDL-like particles after co-sonicaiton with lipids (Fig. 3). Hyd was incapable of solubilizing the lipid vesicles likely because of the short time interval of the experiment (minutes). By cross-linking studies, hyd showed the most oligomerization when dissolved in aqueous buffer (Fig. 2), which may have limited its ability to dissociate into monomers and then interact with the phospholipid vesicles in the relatively short time frame of the study. Simulations clearly show, however, that hyd monomers can bind and penetrate into lipid membranes (Supplementary Figure 9). They could also readily form small HDL-like size particles after co-sonication with lipids (Fig. 3).

Experiments showed that all peptides but neg promoted efflux of cholesterol from BHK-ABCA1 expressing cells, with hyd the most potent (Fig. 5a). In contrast to the vesicle solubilization studies, the cholesterol efflux studies were done over a several hour period, which apparently provided sufficient time for the oligomers of hyd to dissociate and bind to the cell membranes and subsequently promote cholesterol efflux. The next most effective peptide was neu for stimulating ABCA1-dependent cholesterol efflux. This peptide, however, was less active in the vesicle solubilization assay, indicating that this *in vitro* test for assessing the detergent-like properties of peptides does not accurately reflect all of the different steps involved in cellular cholesterol efflux, as has been previously shown for other apoA-I mimetic peptides^36^. Pos was also able to promote cholesterol efflux from cells but was much less potent than the other two active peptides. Similar to the vesicle solubilization studies, neg was completely inactive. This peptide unlike the other peptides was also unable to be reconstituted into an HDL-like particle when combined with phospholipids (Fig. 3a). Similar results were found when the peptides were tested for cholesterol efflux from J774 cells expressing ABCA1 but neu was relatively less active in J774 cells compared to BHK-ABCA1 cells (Fig. 5a). The reason for this is not known, but besides ABCA1, J774 cells can efflux cholesterol by multiple mechanisms^43^ which may account for the different efflux results in the two cell lines.

We also tested the effect of the addition of peptides to plasma on cholesterol efflux (Fig. 5d). All the peptides except neg were able to form pre-beta HDL and cause a small increase in plasma cholesterol efflux. Again this probably relates to the relatively poor ability of neg to bind to lipids., Neg also inhibited cholesterol efflux when added to plasma, although it did not block pre-beta HDL formation when mixed with pos (Fig. 5c) or cholesterol efflux from BHK-ABCA1 cells when mixed with pos (Supplementary Figure 8). We chose pos to do the mixing studies with neg because based on the MD simulation studies we hypothesized that a favorable charge interaction may occur between the two peptides that could increase peptide dimerization and enhance cholesterol efflux. Additional studies will be needed to better understand the inhibitory effect of the neg peptide on plasma cholesterol efflux.

All-atom MD simulations provided valuable insights into how the ELKs interact with lipids and promote cholesterol efflux. Simulation of ELKs on a membrane surface (Set 1), which was used to simulate the interaction of the peptides with lipid domains created by ABCA1 on the plasma membrane, showed that all ELKs but neg lie under the phosphate groups. The superficial localization of neg indicates its weak lipid binding affinity and partly explains its inactivity in solubilizing lipids or promoting cholesterol efflux. Simulation of peptides on a membrane edge (Sets 2) demonstrated that all ELKs but pos tend to form dimers. A similar simulation but with a higher density of peptides per edge (Set 3) showed that neu and hyd can arrange next to each other to form oligomers, whereas pos and neg dimers repel each other and migrate to the head group surface. The optimal coverage of acyl chains with hyd, which is due to fully matched adjacent peptides (Fig. 7), explains its superior ability to stabilize a nanodisc formed after cholesterol efflux. Finally, a long simulation (Set 4) of neu on a nanodisc demonstrated that neu peptides diffuse from the surface to the edge and cover the acyl chains in a picket-fence arrangement on the microsecond time scale. The simulation suggests that when lipid domains are pleated by ABCA1 transporters, the bound peptides rapidly migrate to reduce the hydrophobic cost of the bare acyl chains. It further validated the presumed picket-fence arrangement of peptides in Sets 2 and 3, and the findings of Set 2 whereby antiparallel neu dimers were more favored than parallel ones.

Overall, the results of this study are consistent with a model whereby cholesterol efflux of amphipathic peptides depends on their ability to bind lipids and then to form a stable HDL-like scaffold. The former concerns the pre-efflux event when peptides bind to a lipid domain created by ABCA1, and the latter concerns the efflux and post-efflux events when peptides remodel the pleated lipid domain to form a nanodisc^44^. The three ELKs (hyd, neu and pos) that demonstrated deep insertion in MD simulations, an indication of high lipid binding affinity, all have large hydrophobic faces (subtending angles > 140°), as well as lysine residues at the hydrophobic/hydrophilic boundary of the amphipathic helix. Thus, hydrophobic residues maintain hydrophobic contact with acyl chains, while lysine residues “snorkel” to interact with phosphate groups. Note that a very large hydrophobic face, such as that of hyd (subtending angle = 200°), can potentially reduce the rate of lipid binding due to oligomerization of lipid-free peptides, as observed in the vesicle solubilization studies (Fig. 4). The ability of ELKs to form a stable HDL-like scaffold also mimics the structural role of apoA-I. While there is a consensus that apoA-I forms a double belt in nascent HDL, microseconds-long MD simulation revealed that the neu peptide, and presumably the other active peptides, form a picket-fence arrangement. Such an arrangement of peptides should stabilize the nanodisc formed after cholesterol efflux, and therefore, peptides with this property should show superior cholesterol efflux ability. Peptide dimerization, however, may not be sufficient. Neg, for example, initially dimerizes, but the dimers eventually repel each other in our simulation (Fig. 7 and Supplementary Figure 10) and failed to form a stable scaffold. Neu or hyd, in contrast, can simultaneously form salt bridges with two neighbors (Fig. 7) and form a stable peptide oligomer. In summary, the present study showed the importance of Type A amphipathic helices with an optimal hydrophobic face for lipid binding and their ability to oligomerize for nanodisc stabilization. These insights inform the future design of apoA-I mimetic peptides.

## Methods

All experiments with human samples were performed in accordance with relevant guidelines and regulations. Human blood was drawn from healthy volunteers with informed consent and with the approval of the Blood Bank at the National Heart, Lung, and Blood Institute (approval no. 99-CC-0168).

### Peptide Synthesis

Four ELKs were designed and synthesized by a solid-phase procedure, using FMOC amino acids, on a Biosearch 9600 peptide synthesizer (Milligen, Bedford, MA). Peptides were purified to more than 95% purity by reverse-phase HPLC on an Aquapore RP-300 column (Perkin Elmer, Waltham, MA).

### Circular Dichroism (CD) spectroscopy

UV circular dichroism spectra were obtained on a Jasco J715 spectropolarimeter at 24 °C to measure the helicity of peptides in 1% acetonitrile (ACN) and 10% trifluoroethanol (TFE). Stock peptides were diluted to 0.1 mg/ml in PBS, pH 7.4, and loaded into a quartz cuvette (d ¼ 0.2 cm path length) and the CD spectra from 185 to 240 nm were recorded. The instrument response time was 0.5 s, with a bandwidth of 1 nm and a scanning speed of 50 nm/min. The helix was identified by its characteristic CD spectrum which has a minima at 198 nm and a maxima at 218 nm^45^. Data were normalized by calculating the mean residue ellipticity using a mean residual weight of 121^46^. Helical estimates were determined from the mean residual ellipticity at 222 nm^47^.

### Cross-linking of peptides

Cross-linking of the ELKs was performed by using bis(sulfosuccinimidyl) suberate (BS^3^) cross-linker (Thermo Scientific, Rockford, IL, cat# 21586). BS^3^ was dissolved in PB at 50 mM immediately before use. Cross-linker was added to the peptide at 20-fold molar excess and incubated at room temperature for 30 mins. The reaction was quenched with 20 mM Tris buffer for 15 minutes at room temperature. Reaction products were analyzed by SDS-PAGE in a 12% acrylamide bis-tris gel followed by Coomassie staining for 15 minutes.

### Solubilization of lipid vesicles by peptides and determination of vesicle sizes

Detergenic ability of peptides was measured by a method described previously^36,46^. Briefly, dimyristoyl phosphatidyl choline (DMPC) vesicles (1 mg/ml) were prepared by re-suspension of dried DMPC in PBS and vortexing for 5 minutes. Changes in light scattering upon addition of peptides (final concentration of 100 ng/ml) were recorded at 24 °C every 5 seconds for 50 mins at 432 nm, with shaking (fast setting) in a Victor3 microplate reader (Perkin Elmer, Waltham, MA). The effect of the peptides was compared with a negative control solution containing only PBS and expressed as the percentage of area under the curve (AUC). For natural lipids, components of the commonly found phospholipids in the membranes (bovine liver phosphatidylcholine (55%, w/v), porcine brain spingomyelin (12%), egg lysophosphatidylcholine (5%), bovine liver phosphatidylethanolamine (10%), porcine brain phosphatidylserine (8%), bovine liver phosphatidylinositol (10%)) were mixed and suspended in a solution of methanol, chloroform and water (20:9:1)^36^. The mix was dried with nitrogen gas flow and re-suspended in PBS followed by vortexing for 5 minutes.

For determination of sizes of vesicles formed by the peptides, lipids were supplemented with 0.5% rhodamine conjugated phosphatidylethanolamine. The reaction of lipids and ELKs was set up as described above with an incubation period of 2 hours at room temperature in a dark chamber with intermittent shaking. The particles were separated by electrophoresis in a native 1 dimension TBE gel. The gel was scanned in a Typhoon Scanner.

### Electron microscopy

The peptide and POPC were mixed at 1:6.25 neu:POPC molar ratio and cholesterol was added to this mix at 10:1 POPC:chol molar ratio. Chloroform solution of cholesterol was dried under nitrogen gas. The entire mixture was solubilized in glacial acetic acid and dried by lyophilization. The dried vesicles were re-suspended in PBS, sonicated and filtered through a 0.22 micron filter. The final ratio of peptide and lipid in the particles was determined by performing BCA and choline hydroxylase assays and was to be 1:6.25. TEM images were obtained using a JEM 1200EX electron microscope (JEOL, USA) equipped with an AMT XR-60 digital camera (Advanced Microscopy Techniques Corp.). Vesicle samples were deposited on carbon film-coated 400 mesh copper grids (Electron Microscopy Sciences) and dried for 1 min. The samples were negatively stained with 1% uranyl acetate solution; the grids were blotted with tissue and dried before TEM observation.

### Cholesterol efflux assay

The protocol for *in vitro* cholesterol efflux assay was described previously^18,30^. Briefly, stably transfected BHK cells with the human ABCA1 transporter gene under the mifepristone-inducible promoter or mock-transfected BHK cells and J774 macrophages were treated with/without 0.3 mmol/L 8-(4-chlorophenylthio)-cyclic AMP (Sigma, Darmstadt, Germany) to upregulate the ABCA1 transporter and were incubated with 1 μCi/ml of [^3^H]-cholesterol (Perkin Elmer, Boston, MA, USA) in serum free DMEM. After 24 h incubation, the media was replaced with DMEM containing ELKs or PBS as vehicle control. After 18 h incubation, cellular medium was collected and filtered through a Unifilter 25 mm polypropylene filter (Whatman Inc., Florham Park, NJ) and cells attached to the bottom were lysed in 0.4 ml of 0.1% SDS and 0.1 M NaOH. Radioactive counts in medium and cell lysates were measured by liquid scintillation counting on a Perkin Elmer MicroBeta 1450 scintillation counter. Cholesterol efflux was expressed as the percent of total [^3^H]-cholesterol transferred from cells to medium. Non-specific efflux (i.e. the efflux in the absence of an acceptor) was subtracted. Efflux of apoB-depleted plasma before and after peptide addition was performed as above, using a final 1% (v/v) concentration of plasma.

### Plasma HDL remodeling

Plasma pool of 10 normolipidemic subjects was treated with 20% polyethylene glycol (MW 8000, Sigma-Aldrich, St. Louis, MO) for 15 min at room temperature to remove apoB-containing lipoproteins. The peptide was mixed with plasma at 0.5 mg/ml and incubated at 37 °C with shaking at 300 rpm for 1 hour. 1D native gels (nondenaturing gradient gels) were used to separate intact HDL particles by size by a method adapted previously^48^. After incubating plasma with peptides, the plasma-peptide mixture was mixed with an equal volume of Novex Tris–Glycine Native Sample Buffer (2x) and immediately loaded onto Novex™ 10–20% Tris-Glycine WedgeWell™ minigels, 1.0 mm thick (Life Technologies). The running buffer was 1x Novex Tris–Glycine Native Running Buffer. Gels were run at 100 V for 5.5 hours at room temperature and transferred to PVDF transfer membrane (0.45 µm) (Life Technologies). The running buffer was^48^. After blocking, the membrane was incubated with anti-human apoA-I antibody, preconjugated to HRP (Meridian Life Sciences, Memphis, TN) washed, and detected with Western Lightning (Perkin-Elmer, Waltham, MA).

### Red cell hemolysis assay

Human red blood cells (RBC) were washed 5 times with PBS (pH 7.4). Final concentration of RBC was approximately 4% hematocrit in PBS. Cell toxicity of peptides was tested by incubating RBC for 2 h at room temperature, with the indicated concentration of the peptides, followed by centrifugation at 1000 *g* for 5 min and measurement of the absorbance of the supernatant at 450 nm. Triton X-100 (1%) was able to completely lyse the cells and was used as 100% lysis to compare the peptide induced toxicity^36^.

### Simulation details and analyses

As presented in Table 2, four sets of simulations were performed. In Set 1, peptides interacted with lipid head groups, in Set 2 and 3, they interacted with acyl chains of a bilayer slab, and in Set 4, they interacted with a nanodisc (Fig. 6).

In Set 1, four simulations were performed for four ELKs. To set up each simulation, two identical α-helical peptides were generated and placed at *z* = ±20 Å. The CHARMM-GUI interface^49^ built a lipid bilayer between the peptides, with *z* axis along the bilayer normal, and added 50 water molecules per lipid and 150 mM NaCl to the assembly. The systems were simulated at constant pressure (P) and temperature (T) with constant number of particles (N) (NPT ensemble). The pressure was applied semi-isotropically, i.e., the extension of the simulation box in the direction of bilayer normal and bilayer plane could vary independently.

In Sets 2 and 3, a lipid bilayer was first equilibrated with water and 150 mM NaCl. The water molecules and ions were then removed, hence a bilayer slab was obtained with six bare surfaces (two surfaces contain lipid head groups and four surfaces contain acyl chains). Peptides were placed on two edges containing the acyl chains and were oriented along the bilayer normal with their hydrophobic residues facing the acyl chains. Water and ions were then added to surround the assembly from the surfaces containing lipid head groups and peptides. The bilayer was periodic along the remaining two edges. In Set 2, the bilayer was duplicated so the edges containing the peptides were twice as large as the edges interacting with their periodic images. The bilayer consisted of 80 POPCs and 8 cholesterols. 8 peptides were arranged parallel (head-to-head) and antiparallel (head-to-tail) to each other on each edge, 4 per edge. The boxes were cubic and the pressure was applied isotropically, i.e., the box could extend or compresses in all three directions equally and simultaneously (NPT simulation). The systems were hydrated with 88 water molecules per POPC and 150 mM NaCl. Set 3 included 60 POPCs, 6 cholesterols, and 8 peptides. 2 antiparallel dimers (4 peptides) were initially placed on each edge; the dimers were selected from Set 2. The boxes were tetragonal, and the pressure was applied along the direction of bilayer periodicity. The area (A) of the plane perpendicular to the direction of bilayer periodicity was constant (NPAT simulation). This cross-section is shown in Fig. 7c. The systems were hydrated with 135 water molecules per POPC and 150 mM NaCl.

The nanodisc in Set 4 was built using the CHARMM-GUI interface^49^. 24 peptides were then randomly placed adjacent to the disc surfaces, 12 per surface, and 777 water molecules per POPC and 150 mM NaCl were added to the assembly. Peptides were pulled toward the bilayer for approximately 10 ns to associate with its surfaces. Peptides’ helicities were restrained during the pulling process. The simulation was extended for 2 µs on Anton-2 supercomputer.

All systems used CHARMM 36 lipid and protein parameters^50–52^. TIP3P water model^53^ as modified for CHARMM^54^ was used to describe water molecules. The Lennard-Jones (LJ) parameters for the interaction of Na^+^ and Cl^−^ as well as Na^+^ and selected oxygens of lipids and proteins were taken from the CHARMM C36 ion parameters (NBFIX terms)^55–57^.

Specific details of simulations on in-house computers: Systems were simulated and analyzed using CHARMM 39b2^58^. Trajectories were generated with a leapfrog Verlet algorithm with a time step of 1 fs. Temperature and pressure were kept at 310 K and 1 bar using the Nose-Hoover thermostat^59,60^ and Langevin barostat, respectively. The masses of temperature and pressure pistons were 20% and 2% of the systems masses, respectively. Lennard-Jones potentials were terminated at 12 Å, with a smoothing function operating between 8 and 12 Å. Electrostatics were evaluated using particle-mesh Ewald^61^ with approximately 1 grid point per Å, a sixth-order spline interpolation for the complementary error function, a real-space cutoff of 12 Å, and a κ value of 0.32. All bonds to hydrogen atoms were constrained using the SHAKE algorithm^62^.

Specific details of simulations on Anton-2 supercomputer: Trajectories were generated with a multigrator^63^, which separates barostat, thermostat, and Newtonian particle motion updates, with a time step of 2 fs. Temperature and pressure were kept constant using a variant^63^ of the Nosé–Hoover^59^ and the Martyna–Tobias–Klein algorithm^64^. Electrostatic forces were calculated using the u-series method^65^ on a 64 × 64 × 64 mesh for distant calculations. Water molecules and all bond lengths to hydrogen atoms were constrained using M-SHAKE algorithm^66^.

Error bars were calculated by dividing the equilibrated time series into 10 blocks. The error bar equals the standard deviation of means of blocks divided by $\sqrt{10}$.

Hydrophobic moments and hydrophobicities (Table 1) were obtained using MPEx v. 3.2.14^67^ with the Wimley-White hydrophobicity scale based on POPC- water interface^68^. The mean hydrophobic moment is calculated as the vectorial sum of all the hydrophobicity indices, divided by the number of residues. Helical wheel representations of the peptides helixes were generated by HELIQUEST server freely available online^69^.

Peptide height was calculated from the time series of the vertical distance between centers of geometry of backbones of peptides and C2 atoms of oleoyl acyl chains.

Cutoff distances for counting salt bridges were obtained from radial distribution functions as follows: 4.3 Å between nitrogen of amino group of Lys sidechains and carbon of carboxyl group of Glu sidechain; 4.6 Å between nitrogen of amino group of Lys sidechains and carbon of carboxyl group of C-termini; 4.1 Å between carbon of carboxyl group of Glu sidechain and nitrogen of amino group of N-termini; 4.6 Å between nitrogen of amino group of Lys sidechains and phosphorus of POPCs; and 5.8 Å between carbon of carboxyl group of Glu sidechain and nitrogen of POPCs. The cutoff distance for counting hydrophobic contacts was 7.9 Å between carbons of Leu sidechains.

### Statistics

Data are presented as mean ± s.e.m. Either two-tailed unpaired Student’s t-test or one-way ANOVA, using GraphPad Prism version 6.05 software (GraphPad Software, CA, USA), was performed. P values of < 0.05 were considered statistically significant.

### Data availability

Data are available upon request from the corresponding author.

## Electronic supplementary material

Supplemental Information
