# Supplementary material for: Structural properties of apolipoprotein A-I mimetic peptides that promote ABCA1-dependent cholesterol efflux
Source: Sci Rep. 2018 Feb 13;8:2956. doi: 10.1038/s41598-018-20965-2 (PMC5811490; doi:10.1038/s41598-018-20965-2)
Supplement: Supplementary file 1 — Supplemental Information [file 41598_2018_20965_MOESM1_ESM.pdf]

Structural properties of apolipoprotein A-I mimetic peptides that promote ABCA1-dependent cholesterol efflux

Rafique M. Islam[1,2,§], Mohsen Pourmousa[3,§], Denis Sviridov[2], Scott M. Gordon[2], Edward B. Neufeld[2] , Lita A. Freeman[2], B. Scott Perrin Jr.[3], Richard W. Pastor[3*] and Alan T. Remaley[2*]

[1] School of Systems Biology, George Mason University, Fairfax, VA 22030, USA ;

[2]Cardiovascular and Pulmonary Branch, National Heart Lung Blood Institute, National Institutes of Health,Bethesda, MD 20892

[3]Laboratory of Computational Biology, National Heart Lung Blood Institute, National Institutes of Health, Bethesda, MD 20892, USA

[§]Each author contributed equally to the paper.

***Corresponding authors:**

Richard W Pastor

Laboratory of Computational Biology, National Heart Lung Blood Institute, National Institutes of Health, Bethesda, MD 20892, USA, Tel: +1 (301) 435-2035, Fax: +1 (301) 480-6496

(pastorr@nhlbi.nih.gov)

Alan T Remaley

Cardiovascular and Pulmonary Branch, National Heart Lung Blood Institute, National Institutes of Health,Bethesda, MD 20892, USA.Tel: +1 (301) 402-9796, **(**aremaley1@nhlbi.nih.gov**)**

Supplemental Figures and Tables

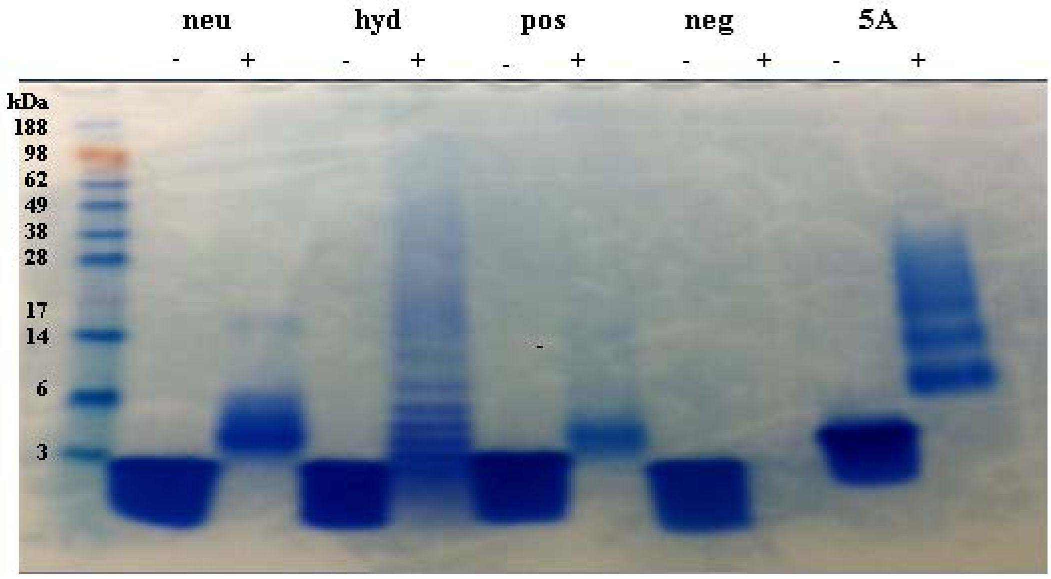

Supplementary figure 1: Uncropped image of cross-linking of ELKs. Cross-linking of ELKs with (+) or without (-) excess BS^3 (bis[sulfosuccinimidyl] suberate) followed by SDS-PAGE (12% Tris-Tricine gel) electrophoresis. A previously tested peptide, 5A, was taken along to serve as positive control.

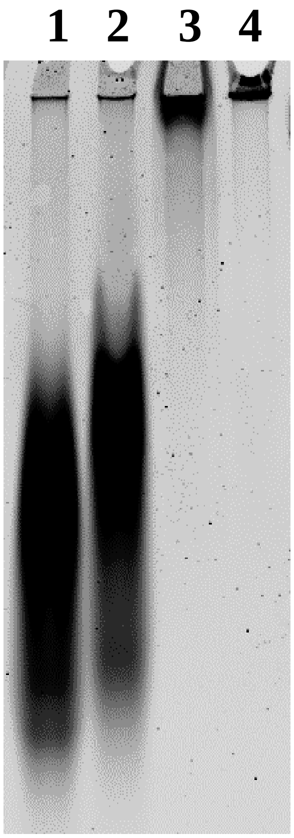

Supplementary figure 2: An uncropped image of ELK- generated particle size with DMPC lipid by native gel electrophoresis. Lipid particles generated by incubation of peptides with DMPC lipid vesicles (lanes 1=neu, 2=hyd, 3=pos, 4=neg). Lipids were suspended in PBS at 1 mg/ml along with 0.5% PE-rhodamine. The vesicles were incubated with 0.5 mg/ml peptides in PBS at room temperature for 2 hours with gentle shaking. The lipid particles formed by peptides were separated in a 1 dimension native TBE gel and scanned using a Typhoon scanner.

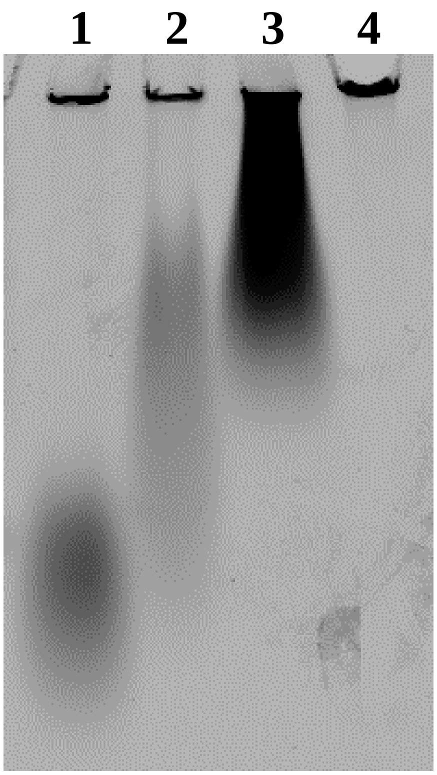

Supplementary figure 3: An uncropped image of ELK- generated particle size with a mixture of natural lipids by native gel electrophoresis. (a) Lipid particles generated by incubation of peptides with a mixture of natural lipids found in cellular membrane (lanes 1=neu, 2=hyd, 3=pos, 4=neg). Lipids were suspended in PBS at 1 mg/ml along with 0.5% PE-rhodamine. The vesicles were incubated with 0.5 mg/ml peptides in PBS at room temperature for 2 hours with gentle shaking. The lipid particles formed by peptides were separated in a 1 dimension native TBE gel and scanned using a Typhoon scanner.

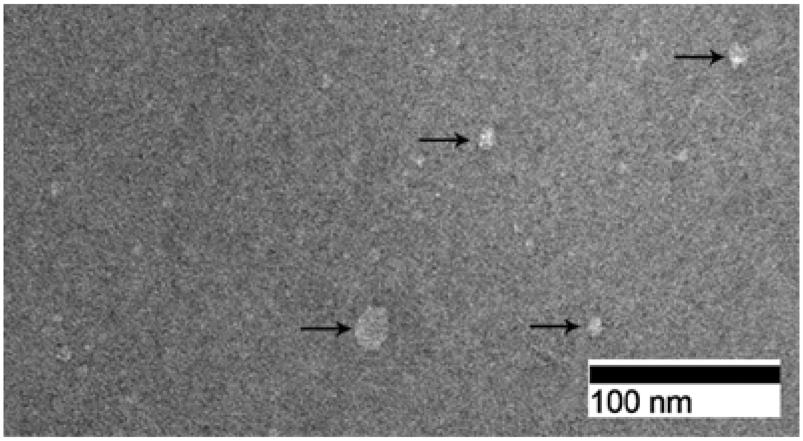

Supplementary figure 4: An uncropped image of ELK-neu peptide generated particle size with POPC by EM. (a) Lipid particles generated by incubation of ELK-neu peptide with POPC lipid vesicles. Particle size distribution after reconstitution of neu with phospholipid and cholesterol (neu:POPC=1:6.25 and cholesterol:POPC=1:10). Mean size of particles is 9.2 nm. Scale 100 nm, magnification 50000.

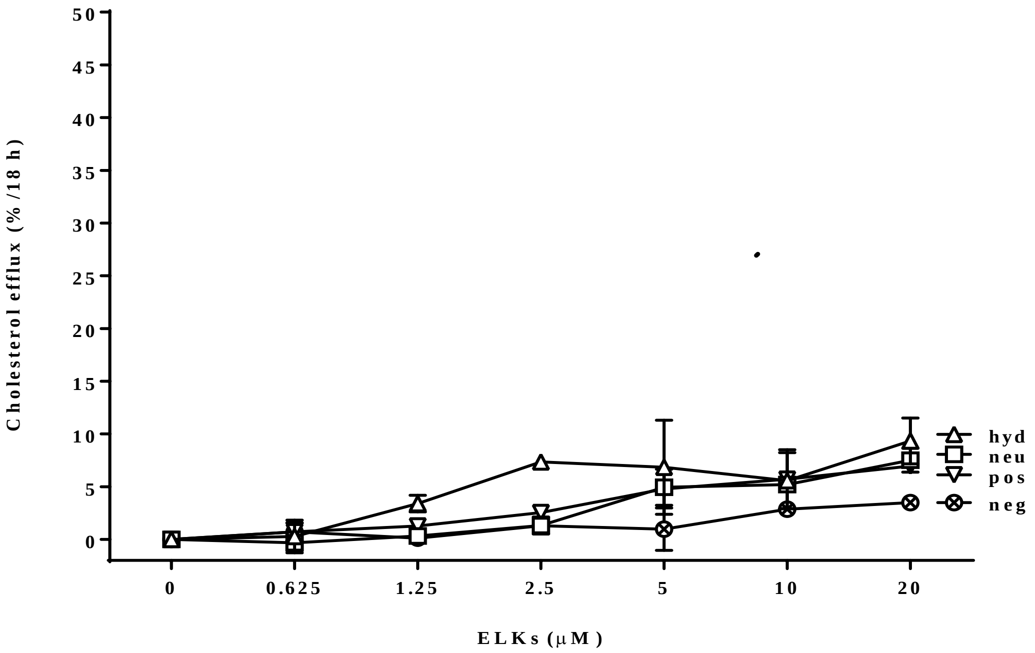

Supplementary figure 5: Efflux assay for ELKs in mock-transfected BHK cells. Cells were treated similarly as ABCA1 expressing BHK cells. Results represent mean ±1SD of triplicates.

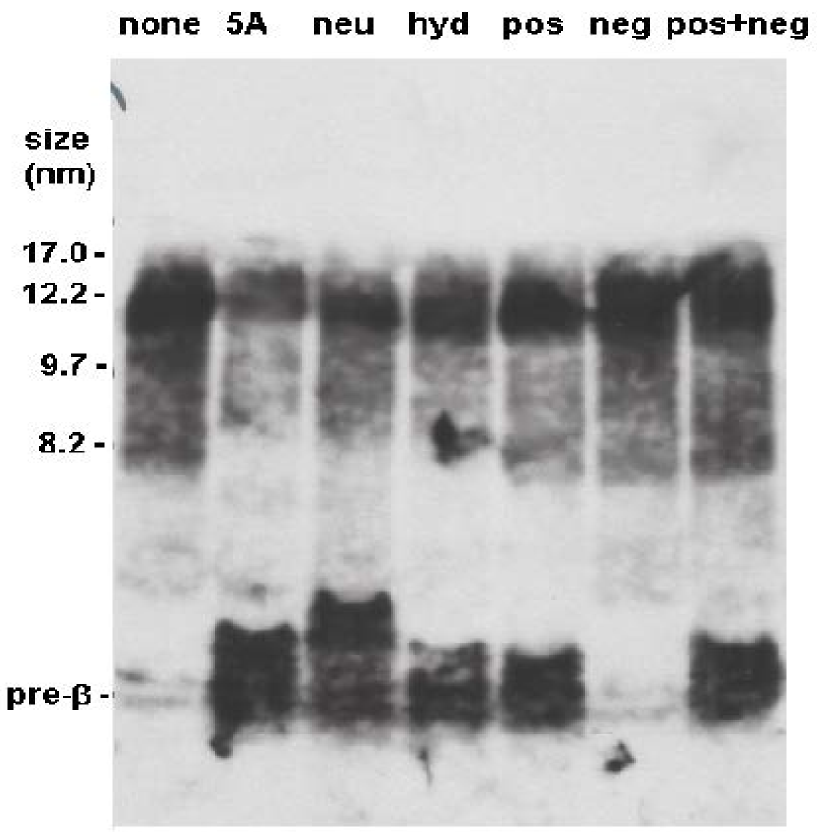

Supplementary figure 6: HDL remodeling in plasma. Uncropped image of the Western blot. Human plasma incubation of ELK peptides causes HDL remodeling. Various level ofsmall pre-β HDLparticles formation was achieved for all but neg peptide.

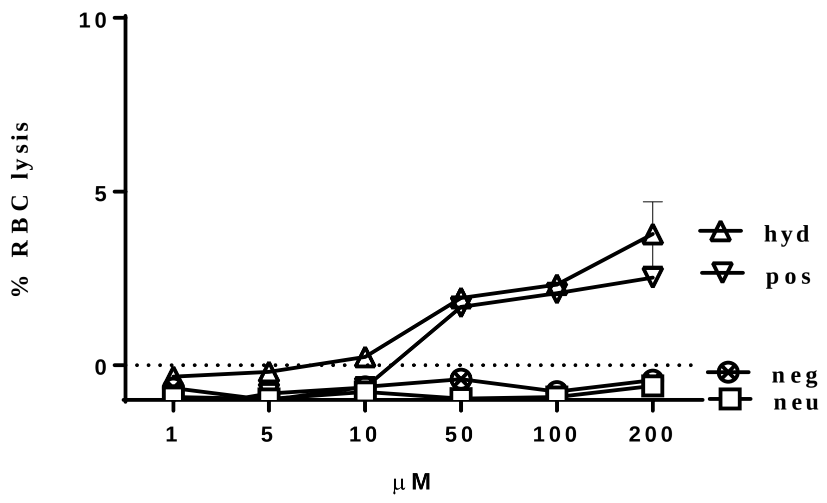

Supplementary figure 7: Red cell hemolysis by peptides. Red blood cell (RBC) lysis experiment was performed, using the indicated concentrations of peptides at room temperature for 2 hours with gentle rotation. Treatment of RBC with 1 % Triton X-100 (μM) saline was used to calculate complete (100%) lysis. Results are expressed as the mean plus minus 1SD of triplicates.

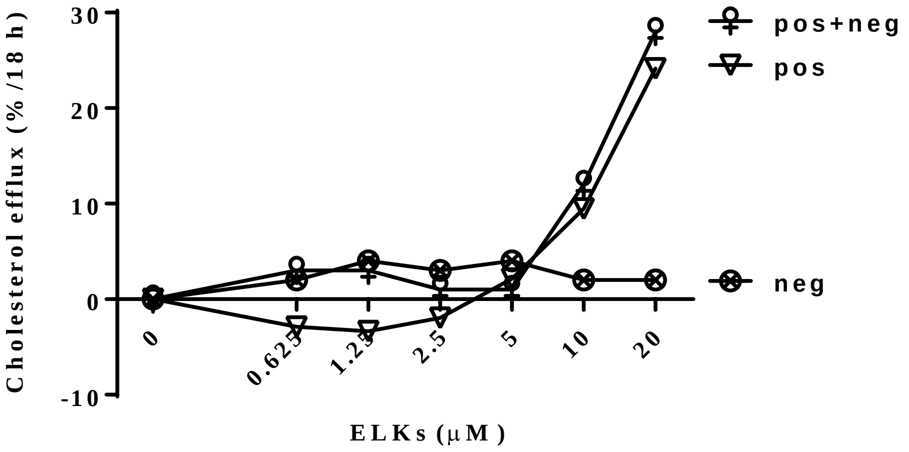

Supplementary figure 8: Efflux assay for pos, neg and combined pos and neg. Cholesterol efflux from ABCA1-BHK cells to ELKs at the indicated concentration on the X-axis plotted**.** Results are expressed as the mean plus minus 1SD of triplicates. For the co-peptide treatment (pos and neg together), the amount of each peptide added is indicated on the X-axis.

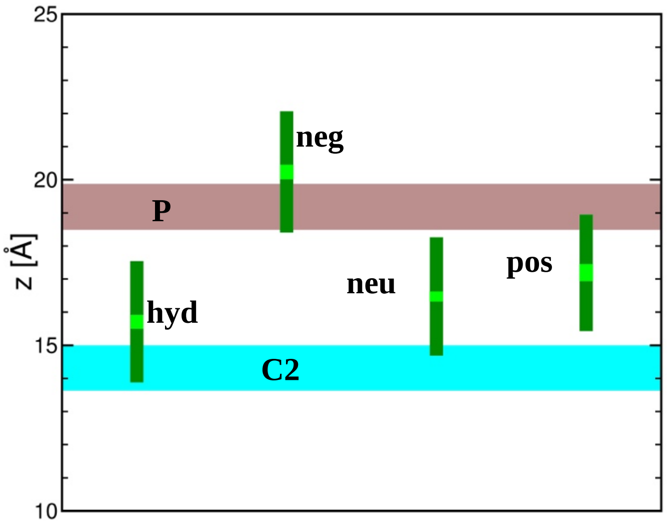

Supplementary figure 9. Insertion depth of ELKs. Center of mass of peptides (green), phosphorus (brown), and C2 atoms of oleoyl acyl chains (light blue) along the bilayer normal (*z*) were obtained from surface-bound simulations (Set 1). Bilayer mid-plane is zero. For each peptide, the length of bar (dark green) is four times standard deviation. For C2 and phosphorus, the width of stripes are two times standard deviations.

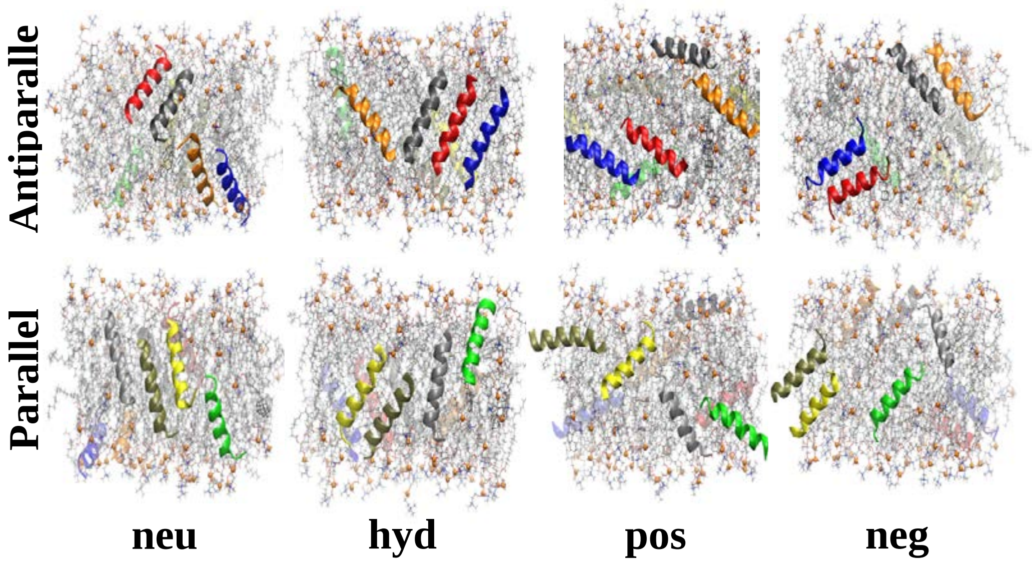

Supplementary figure 10. Dimerization of ELKs on edge of nanodisc patches. Side views of four edge-bound simulations (Set 2, low density) with 80:8:8 POPC:chol:ELK at 500 ns. Each system includes eight identical peptides, shown in colored ribbons, starting from and remaining in antiparallel (top panels) and parallel (bottom panels) orientations on edges of a bilayer slab. Phosphorus of POPC is shown as orange balls. Schematic top-down view is presented in Fig. 6.

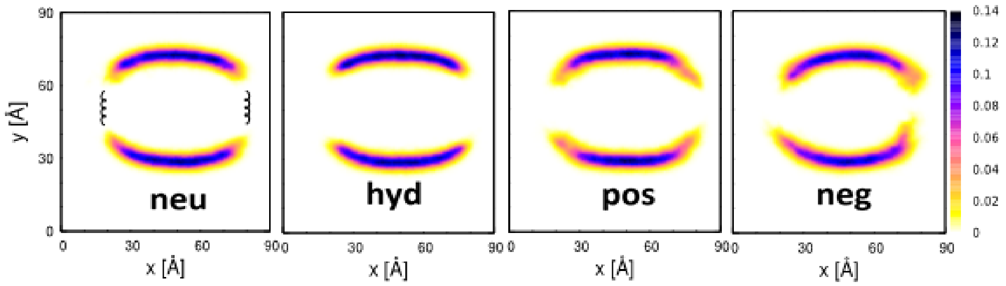

Supplementary figure 11. Density map of Phosphorus of POPCs in Set 3 (high density) edge-bound simulations. Viewed along the direction of bilayer periodicity (see Fig. 6). Peptides are shown schematically in left panel.

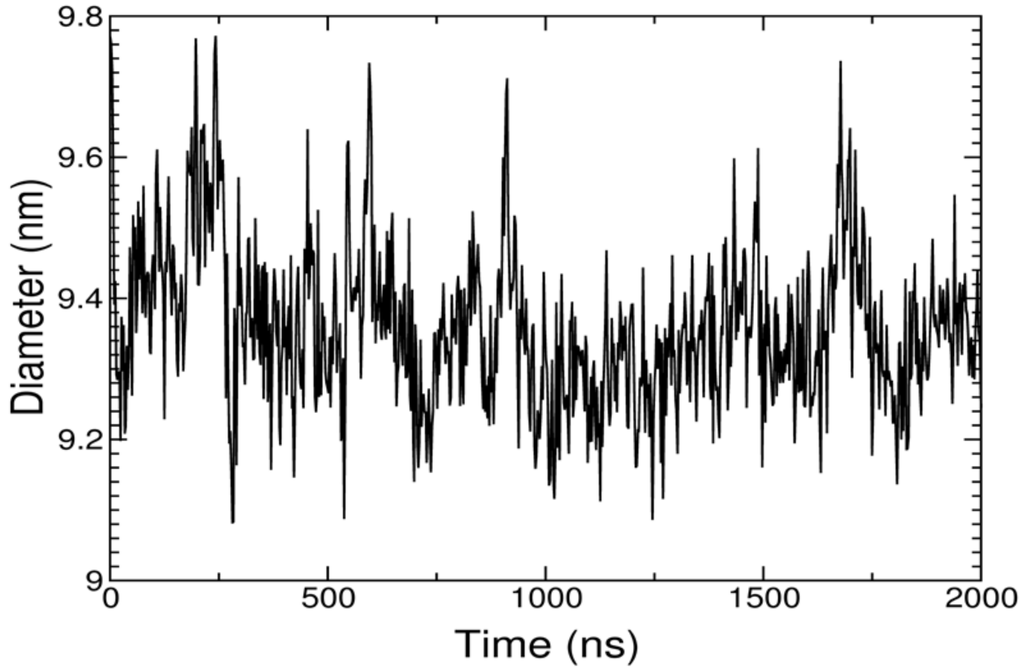

Supplementary figure 12. Time series of the diameter of a discoidal HDL generated by neu (150:15:24 POPC:chol:neu).

Supplementary Table 1. Number of intermolecular salt bridges in Set 2

Interaction	neu				hyd			pos	neg		
	AP1	AP2	P1	P2	AP1	AP2	P1	AP1	AP1	AP2	P1
**salt bridge**	**3.6 (136)**	**3.2 (88)**	**4.0 (117)**	**3.1 (58)**	**0.0 (24)**	**3.7 (214)**	**2.1 (98)**	**2.5 (34)**	**3.8 (202)**	**3.0 (188)**	**3.9 (163)**
**hydrophobic contact**	**7.3 (51)**	**8.5 (19)**	**5.9 (40)**	**5.3 (51)**	**15.7 (41)**	**7.0 (297)**	**9.7 (50)**	**3.3 (12)**	**0.2 (13)**	**0.2 (12)**	**4.3 (19)**

Set 2 (low density) simulations between Lys and Glu sidechains, Lys sidechains and C-termini, and Glu sidechains and N-termini, and hydrophobic contacts between Leu sidechains. Average lifetimes (in ps) are presented in parentheses. Antiparallel and parallel dimers are denoted as AP and P, respectively. Hydrophobic and negative peptides each form one parallel dimer. Positive peptide forms only one antiparallel dimer.
